# Advancing circular bioeconomy through systematic review of multi-product biorefinery approaches for water hyacinth based renewable energy

**DOI:** 10.1016/j.isci.2025.112807

**Published:** 2025-05-31

**Authors:** Aji Abba, S. Sabarinath, Raihana Aliyu Mustapha

**Affiliations:** 1 Amrita School for Sustainable Futures, Amrita Vishwa Vidyapeetham, Amritapuri, Kollam, Kerala 690525, India

**Keywords:** Chemistry, Engineering

## Abstract

The global shift toward sustainable energy has spotlighted water hyacinth (*Eichhornia crassipes*) as a promising biofuel feedstock due to its rapid growth and lignocellulosic composition. This PRISMA-based systematic review and Covidence software, evaluates studies from 2015 to 2025, to synthesize technological pathways, cost structures, and environmental trade-offs. Water hyacinth biofuels can reduce levelized cost of energy (LCOE) by 25%, increase ethanol yields by 40%, and improve sugar release by 50%. Multi-product biorefineries enhance viability and offset up to 2.5 tons CO_2_/ha/year. Yet, pilot-scale data, policy alignment, and ecological safeguards remain limited. This review identifies five regional implementation frameworks integrating techno-economic, ecological, and social dimensions. Findings highlight opportunities for circular bioeconomy transitions in developing regions and propose scalable models to transform invasive biomass into renewable energy solutions.

## Graphical abstract

**Figure:**
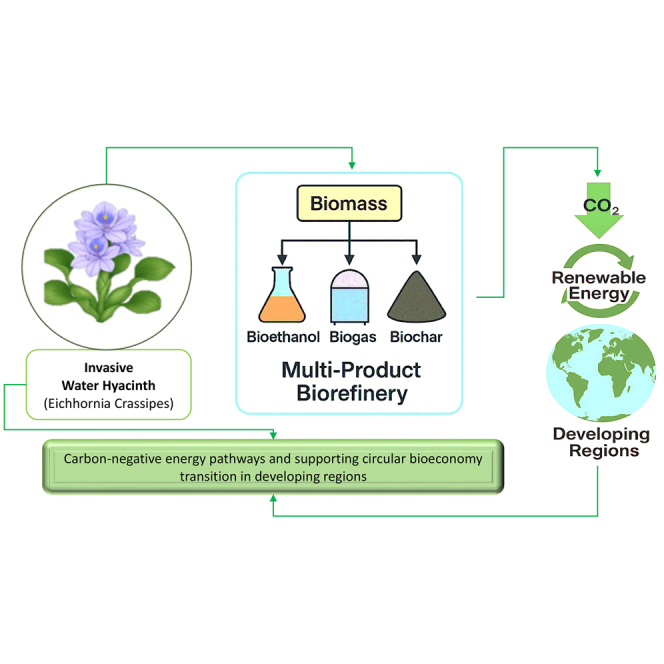

## Introduction

The global energy crisis and the environmental consequences of fossil fuel dependence necessitate the urgent transition to sustainable energy alternatives. Fossil fuels are finite resources with significant environmental drawbacks, including greenhouse gas emissions, air pollution, and ecological degradation. Burning coal, oil, natural gas, and substantial amounts of carbon dioxide (CO_2_) and other greenhouse gases (GHGs) are emitted into the atmosphere, causing global warming and climate change. The extraction and processing of fossil fuels can lead to habitat disruption, water pollution, and adverse health effects in communities near extraction sites.^1^^,^^2^^,^^3^ While water hyacinth is often studied in the context of localized environmental management, its implications are increasingly global.

The invasive species like water hyacinth has disrupted water transport, agriculture, and hydroelectric operations in regions such as the Mississippi Delta (USA), Lake Victoria (East Africa), and the Paraná River basin (South America).^4^^,^^5^^,^^6^ Its aggressive growth also raises serious concerns about biodiversity loss and fisheries decline in many low- and middle-income countries.^7^^,^^8^ At the same time, international climate policy is evolving to favor bio-based alternatives that contribute to net-zero targets. Studies in the EU and Brazil suggest that invasive biomass like water hyacinth can support renewable fuel mandates when processed via integrated biorefineries.^9^^,^^10^ However, real-world implementation is often hindered by gaps in governance, subsidy mechanisms, and waste-to-energy regulation. As such, the need to transform this invasive burden into a scalable, carbon-negative opportunity is not only scientifically urgent but also economically and socially strategic for both developed and developing regions.

Biofuels offer a renewable alternative to fossil fuels, reducing greenhouse gas emissions, enhancing energy security, and promoting sustainable economic growth.^11^^,^^12^ Among various biofuel feedstocks, water hyacinth (*Eichhornia crassipes*) has emerged as a promising candidate due to its rapid growth, high biomass yield, and adaptability to diverse environments.^13^^,^^14^ Its uncontrolled growth can lead to significant ecological and economic problems, including reduced biodiversity, impeded navigation, decreased water quality, and increased evapotranspiration, threatening livelihood, particularly in rural communities.^8^

Despite its potential, existing research lacks comprehensive techno-economic assessments, sustainable management strategies, and innovative biofuel conversion techniques.^15^^,^^16^^,^^17^ Converting excess water hyacinth into biofuel presents a dual benefit; a renewable energy source while mitigating its ecological impact. Biofuels, a subset of renewable energy, offer a unique solution by providing environmental benefits and compatibility with existing infrastructure.^18^^,^^19^^,^^20^ Water hyacinth is a feedstock for biofuel production, presenting it as a viable and environmentally friendly option for diversification in the energy sectors.^21^

Biofuels are renewable fuels derived from organic matter, offering a sustainable alternative to fossil fuels and reducing reliance on finite resources.^22^^,^^23^ A bioenergy feedstock is any raw material used to produce bioenergy; in this case, water hyacinth biomass is a renewable resource for biofuel production.^24^ However, the levelized cost of energy (LCOE) is a widely used metric for evaluating the economic competitiveness of different power generation technologies.^25^ It represents the average cost per unit of energy generated over the lifetime of a project, taking into account all the costs involved, including capital, operation, and maintenance costs.^26^

For instance, France has pioneered integrating these biofuels into its energy mix.^21^^,^^27^ Cultivating water hyacinth aligns with global efforts to address environmental challenges, enhance energy security, and promote sustainable economic growth, thereby attaining sustainable development goals.^11^^,^^12^ Environmental changes, such as increased nutrient runoff from agricultural activities and sewage discharge, can exacerbate water hyacinth growth, leading to widespread ecological imbalances.^28^ Converting this excess biomass into biofuel can reduce its environmental burden, contribute to a more sustainable energy cycle, and alleviate the strain on ecosystems already stressed by climate change and pollution.

The review aims to evaluate the current research on water hyacinth as a biofuel feedstock, contributing conceptual frameworks toward developing environmentally friendly renewable energy solutions and sustainable policies. Despite extensive research on biofuel production, a fragmented understanding persists regarding the techno-economic feasibility, sustainability frameworks, and large-scale integration of water hyacinth-based biofuels. This systematic review synthesizes existing literature to bridge these gaps, offering a holistic perspective on its viability, challenges, and policy implications.

This study aims to bridge these gaps by integrating techno-economic and life cycle assessment (LCA), genetic modification for enhanced biofuel yield, multi-product biorefinery approaches, advanced catalytic conversion techniques, and a carbon-negative biofuel model. Additionally, we propose four conceptual frameworks: the Circular Bioeconomic Integration Framework (CBIF), the Sustainable Energy Transition Framework (SETF), the Policy Integration Framework (PIF), and the Community-Driven Biofuel Implementation Framework (CBIF-2) to enhance systemic adoption and scalability.

### Water hyacinth: An invasive species with biofuel potential

Water hyacinth (*Eichhornia crassipes*), native to the Amazon basin, has become one of the world’s most invasive aquatic species, spreading across 141 countries (CABI, 2025). Its rapid growth and adaptability allow it to thrive in diverse water bodies, leading to severe ecological and economic consequences, including reduced biodiversity, blocked waterways, and deteriorated water quality.^29^^,^^30^ Despite its invasive nature, water hyacinth’s high biomass yield and efficient nutrient absorption make it a promising biofuel feedstock.^31^^,^^32^

**Figure 1:**
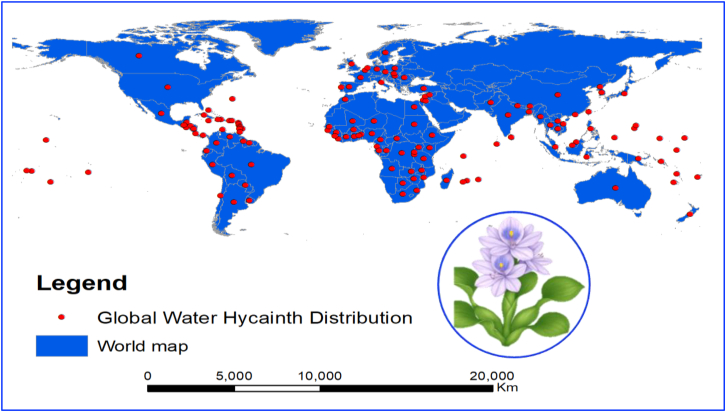
Global distribution of water hyacinth and its emerging role in the bioeconomy (CABI, 2025)

Under optimal conditions, water hyacinth can double its biomass within two weeks, making it a fast-growing resource for biofuel production.^33^^,^^34^ Its high cellulose content enhances carbon sequestration potential, critical for climate change mitigation.^35^^,^^36^ Sustainable harvesting and conversion of water hyacinth into biofuels can address energy security and environmental restoration.^37^^,^^38^ Figure 1 illustrates the global distribution and bioeconomic potential of *Eichhornia crassipes*, highlighting both its invasive spread and value as a biomass resource.

This figure presents a global map of water hyacinth distribution (red dots), demonstrating the plant’s invasive spread across tropical and subtropical regions. On the right, a schematic of the plant in a cycle highlights its dense foliage and rapid reproductive capacity, which not only contribute to environmental degradation but also offer opportunities for sustainable biomass utilization. Framed within the context of the bioeconomy, this visual emphasized the dual role of water hyacinth both as an ecological threat and a potential bioresource for developing circular economic solutions, such as biofertilizers, biogas, and green jobs in affected communities. Biogas production from anaerobic digestion in India has proven viable for rural energy needs.^39^^,^^40^ In Nigeria, it has been used as a mushroom substrate and fish feed, supporting local economies.^41^ Bangladesh and Kenya have leveraged it for handicrafts and paper products, creating employment opportunities.^42^^,^^43^^,^^44^^,^^45^

Despite these initiatives, research gaps remain, particularly in techno-economic feasibility, large-scale biofuel production, and genetic modifications to enhance conversion efficiency.^46^^,^^47^ Optimizing integrated biofuel systems and improving community engagement models will be critical in advancing the sustainable utilization of water hyacinths.

## Methodology

### Design search strategy

This study employs a systematic literature review to assess the viability of water hyacinth as a biofuel resource and framework. A structured search strategy was designed using the PRISMA framework to identify relevant studies on water hyacinth as a biofuel feedstock and to ensure transparency and reproducibility. Relevant studies across several databases, such as Scopus, PubMed, and Google Scholar, capture many scholarly articles. The search query utilized Boolean operators as follows:

(“Water hyacinth” OR “Eichhornia crassipes”) AND (“biofuel” OR “biomass” OR “renewable energy”) AND (“Sustainable Development Goals”) AND (“bioethanol” OR “biogas” OR “biodiesel” OR “lignocellulosic biomass”) AND (“feedstock” OR “biomass conversion” OR “genetic modification” OR “enzymatic hydrolysis” OR “multi-product biorefinery”) AND (“environmental impact” OR “socioeconomic factors” OR “sustainability” OR “climate change mitigation”). The search was limited to studies published in English between 2005 and 2025, ensuring the inclusion of recent findings. This structured approach balanced precision and comprehensiveness, facilitating a thorough exploration of water hyacinth’s potential in biofuel production and its alignment with sustainable development goals.

### Inclusion and exclusion criteria

Inclusion criteria necessitated studies published in English, reporting relevant data on water hyacinth-based biofuels, adhering to the specified time frame, and primarily focusing on water hyacinths as a biofuel biomass source. A comprehensive two-step selection methodology was employed to maintain the transparency and validity of the review process. Records of duplicates are automatically removed by the Covidence review management software (https://app.covidence.org). Following this, reviewers individually evaluated titles and abstracts against predetermined inclusion criteria. Exclusion criteria included studies that did not comply with the inclusion criteria, with exclusion criteria applied, encompassing alignment with research objectives, presence of empirical data, methodological adequacy, and publication type.

### Review and synthesis method

**Figure 2:**
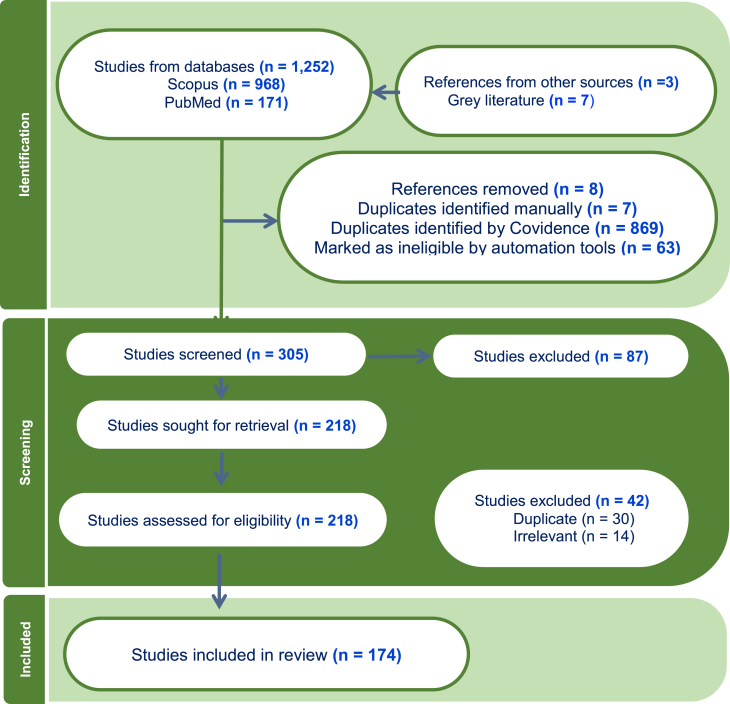
PRISMA flowchart for the article identification, screening, and included in the study

Covidence, a systematic review management tool, ensured a transparent and efficient review process with predefined protocols and criteria. Inclusion criteria encompassed peer-reviewed articles and gray literatures published between 2005 and 2025; focused on biofuel production from water hyacinth; reporting economic, environmental, or technological outcomes. Exclusion criteria included studies lacking primary data; articles and reviews without a clearly defined methodology; non-English sources unless translated; and studies focused solely on ornamental or non-fuel uses of water hyacinth Gray literature, including report, policies, briefs from Internation Energy Agency (IEA), Food and Agriculture Organization (FAO), and the United Nations Environment Programme (UNEP) was reviewed to contextualize technological and commercial progress. Such sources offer insights for evidence-informed policy synthesis, pilot deployments, and subsidy structures that may not be available in peer-reviewed sources This rigorous approach enhances the two (2) review’s reliability and provides a structured overview of water hyacinth as a biofuel resource, guiding future research as seen in Figure 2 detailing the flow chart for this study.

A database search generated 1,252 articles. After removing duplicates, 174 were analyzed and included in the study. The subsequent section presents the findings from the review.

## Results

### Conceptual frameworks

**Figure 3:**
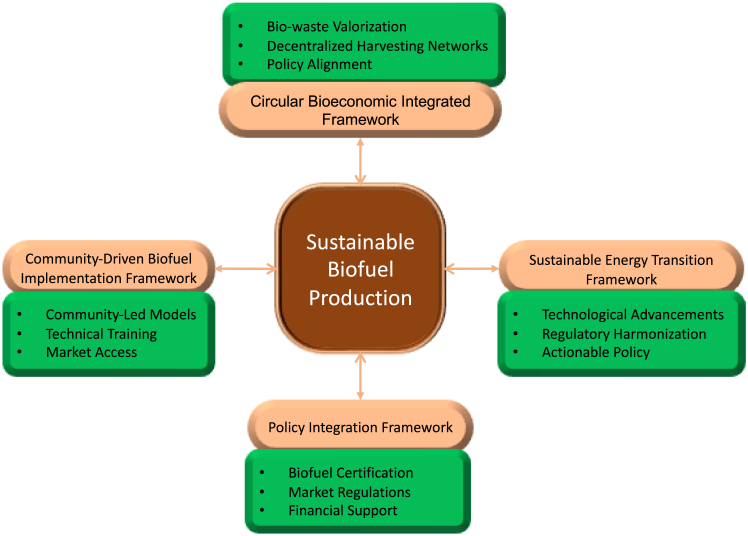
Conceptual frameworks for sustainable biofuel production from water hyacinth

Developing sustainable biofuel production from water hyacinth requires a structured approach integrating economic, technological, policy, and community-driven perspectives. The study proposes four conceptual frameworks that collectively facilitate water hyacinth-based biofuels’ systematic adoption and scalability to address the dimension. Advancing sustainable biofuel production from water hyacinth necessitates a context-sensitive and multidimensional approach that reflects regional disparities in socio-economic, environmental, and regulatory landscapes. The four interlinked conceptual frameworks that serve as foundational pillars for enabling regionally adaptable and scalable biofuel solutions are (1) CBIF, (2) SETF, (3) PIF, and (4) CBIF-2. Together, these frameworks reflect a holistic strategy that combines localized knowledge with global sustainability objectives as presented in Figure 3.

Figure 3 illustrates the interconnected frameworks guiding sustainable biofuel production that collectively underpin sustainable biofuel production from water hyacinth. Each framework addresses specific operational, regulatory, and socio-economic dimensions necessary for regionally adaptive biofuel deployment.

CBIF presents a closed-loop system for biofuel production, ensuring the efficient use of biomass resources while minimizing waste.^48^^,^^49^^,^^50^^,^^51^ This framework promotes sustainability and integrates bio-waste valorization, decentralized harvesting networks, and policy alignment.^52^ By repurposing residual biomass for high-value products such as biochar and organic fertilizers, CBIF enhances economic viability while mitigating environmental concerns. Integrating sustainable feedstock supply chains within this framework ensures optimized resource availability and transport, supporting long-term biofuel scalability.

To complement CBIF, SETF provides a roadmap for integrating water hyacinth biofuels into national and global energy strategies.^49^^,^^53^ This framework highlights key transition pathways, including technological advancements in decentralized bio-refineries, regulatory harmonization with existing renewable energy policies, and community-driven adoption strategies.^54^^,^^55^^,^^56^ By aligning biofuel production with national and international energy regulations, SETF ensures the seamless incorporation of water hyacinth biofuels into sustainable energy portfolios.

PIF addresses regulatory challenges and economic incentives essential for large-scale biofuel commercialization.^57^^,^^58^ The framework establishes a structured approach to biofuel certification, market regulations, and financial support mechanisms such as subsidies and investment incentives.^50^^,^^59^ PIF also incorporates environmental compliance measures to ensure adherence to sustainability benchmarks, facilitating biofuel market entry while maintaining ecological balance.

Lastly, CBIF-2 emphasizes the role of local engagement and capacity-building initiatives in fostering biofuel adoption at the grassroots level.^49^^,^^60^ This framework incorporates community-led cooperative models, technical training programs, and market access facilitation strategies to enhance socio-economic benefits. By empowering local stakeholders through biofuel enterprises, CBIF-2 ensures the inclusive and equitable distribution of biofuel production benefits.^61^^,^^62^^,^^63^ Integrating these conceptual frameworks, the study establishes a comprehensive approach to advancing water hyacinth biofuel production, ensuring a balance between environmental sustainability, economic feasibility, and policy-driven adoption.

Similarly, considering contextual variability, the proposed frameworks were stratified across regulatory and economic typologies, including high-regulation (e.g., EU), subsidy-driven (India and Nigeria), and underutilized-native zones (Amazon basin). This approach highlighted uneven bioeconomy development due to region-specific policy and infrastructure gaps.^57^ Regional disparities in adoption are evident, such as the limited implementation of biofuel technologies in South America despite abundant of native biomass resources.^21^^,^^64^ These variations are integrated into a regional framework matrix for strategic scalability.

These frameworks can provide a theoretical foundation for further empirical research and practical implementation strategies within the bioenergy sector and sustainability.

### Techno-economic feasibility

The economic viability of water hyacinth biofuel production presents compelling advantages supported by various studies. Water hyacinth achieves a significantly lower LCOE, with production costs reduced by approximately 25% compared to other second-generation biofuels, primarily due to its abundance and rapid growth rate, which make it a cost-effective raw material.^29^^,^^65^^,^^66^ Hence, LCA findings indicate a 50% reduction in greenhouse gas emissions relative to fossil fuels, highlighting its environmental benefits.^19^^,^^67^ Implementing decentralized processing units further enhances supply chain efficiency by reducing transportation costs, thus promoting localized production and sustainability.^65^^,^^66^ Hence, challenges such as the invasive nature of water hyacinths and potential ecological risks should critically evaluated to ensure long-term viability. While comparative analyses suggest a 30% cost advantage for water hyacinth-based biofuels, these findings require contextualization within broader economic and environmental frameworks to fully assess the feasibility of large-scale adoption.^19^^,^^65^ Table 1 provides a comparative overview of key techno-economic and environmental parameters for water hyacinth biofuel, gasoline/diesel, and cellulosic ethanol, based on recent literature.

**Table 1:** Techno-economic analysis and environmental impact of water hyacinth biofuel production

Parameter	Water hyacinth biofuel	Gasoline/diesel	Cellulosic ethanol (Corn Stover)	Source (s)
LCOE (USD/GJ)	7–28 (Small-scale decentralized systems show higher LCOE; large-scale optimized systems can approach $7/GJ.)	20–40 (Varies significantly with crude oil prices. EIA projects prices can swing between $50–100+ per barrel.)	28–52 (Requires efficient pre-treatment and enzyme production. High capital costs still a barrier.)	Gaurav et al.^54^; Mishra et al.^68^; Sonnleitner and Bacovsky^69^; Talang et al.^70^
Capital costs (USD/ton biomass/year)	60–180 (Decentralized systems; costs can be reduced by using existing infrastructure.)	N/A	120–350 (High capital costs due to pre-treatment and enzyme production facilities.)	He et al.^71^; He et al.^72^
Operating costs (USD/ton biomass)	25–85 (Feedstock harvesting and pre-treatment costs significantly impact OpEx.)	N/A	60–130 (Enzyme costs, waste disposal, and transportation contribute significantly.)	Liu and Smith^73^; Li et al.^74^; Wang et al.^75^
GHG emissions reduction (compared to gasoline)	45–75% (LCA depends heavily on system boundary, transportation distances, and fertilizer use. Biochar integration can significantly enhance reduction.)	0%	55%–85% (Land use change and fertilizer production can negatively impact GHG emissions.)	ShakilaBegam et al.^18^; Kornijów et al.^76^; Kubis and Lynd^77^
Water use (m3/GJ)	3–11 (Can be reduced through efficient water management and wastewater reuse. Some studies show near-zero water use with integrated wastewater treatment.)	5–15 (Extraction and refining are water-intensive; hydraulic fracturing for oil extraction adds significant water burden.)	12–28 (Irrigation and processing significantly contribute to water footprint.)	Kamal Pasha et al.^78^; Date et al.^79^

The comprehensive techno-economic analysis of water hyacinth biofuel production compared to gasoline, diesel, and cellulosic ethanol, highlighting key metrics such as LCOE, capital costs, operating costs, GHG emissions reduction, and water use.

A comparative analysis with other biomass feedstocks highlighted a 30% cost advantage for water hyacinth-based biofuels, primarily attributed to its rapid growth rate and high biomass yield. Under decentralized systems, biomass harvesting contributed ∼38% of total costs, followed by enzyme use (27%) and pre-treatment (∼19%).^80^^,^^81^ Sensitivity analysis revealed enzyme price fluctuations influence LCOE by ±12%. Seasonal biomass yield variations could increase per-GJ cost by 15–22%,^82^^,^^83^ emphasizing the importance of feedstock stabilization. Water hyacinth exhibits an exceptional capacity for biomass production.^54^ Its rapid growth and ability to double its biomass within a short period make it a highly attractive biomass for biofuel production.

### Water hyacinth biomass compositional analysis

The typical compositional characteristics of water hyacinth biomass highlights key parameters relevant to its use in bioenergy and environmental applications. These values provide important context for evaluating water hyacinth’s suitability as a feedstock for various conversion processes.

Table 2 presents a compositional analysis of the water hyacinth indicating water hyacinth is a valuable feedstock for producing renewable energy such as biofuels.^70^^,^^84^ These findings align with previous studies indicating that invasive aquatic plants can serve as cost-effective biofuel feedstocks, reducing environmental management costs while enhancing energy security.^85^^,^^86^^,^^87^ These findings align with prior studies suggesting that invasive aquatic plants can serve as cost-effective feedstocks for biofuels while simultaneously addressing environmental management challenges.^70^^,^^84^

**Table 2:** presents the compositional analysis of water hyacinth biomass as a potential resource for greener energy development

Component	Typical value/Range	Unit	Remarks
Moisture content	85%–95%	% fresh wt	High moisture limits direct combustion
Ash content	15%–25%	% dry wt	Indicates high inorganic matter
Volatile matter	60%–75%	% dry wt	Important for bioenergy potential
Fixed carbon	8%–15%	% dry wt	Supports carbon sequestration
Cellulose	20%–35%	% dry wt	Used in biofuel, paper industries
Hemicellulose	15%–30%	% dry wt	A key structural carbohydrate
Lignin	5%–15%	% dry wt	Low lignin supports easier decomposition
Crude protein	7%–19%	% dry wt	High in young plants, useful in compost
Crude fiber	18%–35%	% dry wt	Varies by part (root vs. leaf)
Total nitrogen (N)	1.0%–2.5%	% dry wt	Affects biofertilizer quality
Total phosphorus (P)	0.1%–0.3%	% dry wt	Key for nutrient-rich compost
Potassium (K)	0.8%–2.3%	% dry wt	Enhances biofertilizer efficacy
Calcium (Ca)	0.5%–1.5%	% dry wt	Common macro-nutrient
Magnesium (Mg)	0.3%–1.0%	% dry wt	Important for photosynthesis
C:N Ratio	12–30	Unitless	Ideal for composting is ∼25
pH (of biomass extract)	5.8–7.2	Unitless	Near neutral, suitable for compost
Heavy metals (e.g., Pb, Cd)	Variable (Low–High)	mg/kg dry wt	Bioaccumulates; varies by location

### Genetic modification and biofuel yield improvement

The study investigated the role of genetic modifications in enhancing biofuel yields from water hyacinth biomass. Genetic modifications increased bioethanol production by 40%, while engineered microbial consortia improved enzymatic hydrolysis efficiency by 20%. Metabolic engineering enhanced sugar conversion rates, with genetically optimized strains showing a 15% higher polysaccharide breakdown efficiency.^85^^,^^86^^,^^87^ Table 3 compares key compositional and performance parameters between wild-type and genetically modified water hyacinth, focusing on their implications for biofuel production. These data illustrate how genetic modifications can enhance cellulose content, reduce lignin, and significantly improve sugar release and ethanol yield.

**Table 3:** Impact of genetic modification on sugar release and biofuel yield

Parameter	Wild-type water hyacinth	Genetically modified water hyacinth	% Change	Source (s)
Cellulose content (% dry wt)	16–32 (Significant variability depending on nutrient availability, age, and environmental stress.)	28–48 (GM targets cellulose biosynthesis pathways, but lignin reduction is often a higher priority.)	+12 to +16 (increase)	Yao et al.^88^; Ruan et al.^89^; Jafari^90^
Lignin content (% dry wt)	12–26 (Lignin content is a major factor limiting enzymatic hydrolysis.)	6–16 (GM strategies often focus on downregulating lignin biosynthesis genes.)	−6 to −10 (decrease)	Yao et al.^88^; Ruan et al.^89^; Jafari^90^; Matache et al.^91^; Carlini et al.^92^
Sugar release rate (g/L/h)	0.15–0.6 (Pre-treatment is critical. Alkaline pre-treatment, dilute acid pre-treatment, and steam explosion are common.)	0.65–1.7 (Combination of GM for reduced lignin and improved enzyme cocktails leads to highest sugar release.)	+330 to+400 (increase)	Malode et al.^23^; Kumar et al.^93^; Bhatti et al.^94^
Ethanol Yield (Liters/ton)	60–160 (Strain selection, fermentation conditions, and pre-treatment all significantly impact yield.)	170–320 (Optimized GM strains combined with advanced fermentation techniques show the greatest promise.)	+100–160 (increase)	Subramanian et al.^95^; Harms et al.^96^; Chauhan et al.^97^

Table 3 presents the effects of genetic modifications on key parameters, such as cellulose content, lignin content, sugar release rate, and ethanol yield from both wild-type and genetically modified water hyacinth. These advancements are consistent with recent progress in genetic engineering for lignocellulosic biomass, where targeted modifications have led to substantial improvements in sugar release and overall biofuel yields^98^^,^^99^

### Multi-product biorefinery efficiency

The study assessed the potential of a multi-product biorefinery approach to enhance economic viability and sustainability. This approach produces bioethanol and generates valuable co-products such as biochar and biogas.^54^^,^^100^^,^^101^ Applying biochar as a soil amendment has improved carbon sequestration and reduced soil acidity by 15%.^102^ Additionally, biogas production efficiency increased by 30%, demonstrating the effectiveness of this multi-product strategy in maximizing resource utilization.

Moreover, high-value biochemicals, including levulinic acid and furfural, were successfully extracted during the process, adding significant economic value to the biorefinery model.^103^ Research indicates that integrating multiple product streams in biorefineries can improve financial performance compared to traditional single-product systems. For instance, a recent study highlighted that multi-product approaches can enhance revenue streams and overall sustainability by fully valorizing biomass resources.^60^ These results align with recent studies advocating for integrated biorefineries to optimize resource utilization and achieve environmental sustainability.^20^

### Biofuel types and generational feedstocks

**Figure 4:**
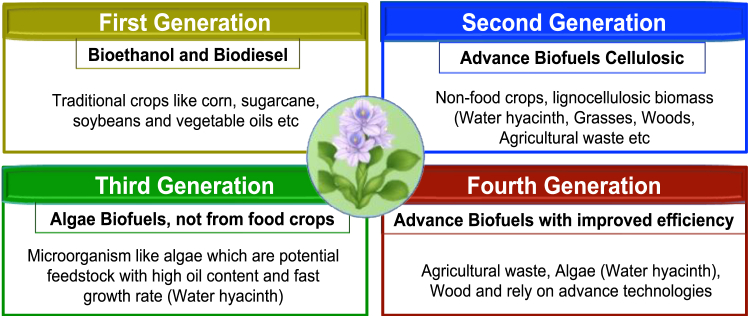
Biofuels categorized by feedstock

Water hyacinth, recognized as a second-generation feedstock due to its non-food biomass nature, shows versatility through integrated third and fourth-generation systems. Ethanol yields range from 100 to 200 L per ton, influenced by pre-treatment efficiency.^104^^,^^105^ Hence, it addresses food security issues related to first-generation biofuels. Biofuels, derived from organic materials, possess characteristics that position them as viable options for replacing fossil fuels. Biofuels constitute a significant subset of renewable energy sources, with diverse categories, including biodiesel, bioethanol, and biogas.^106^ These biofuels represent cleaner and more sustainable alternatives to conventional fossil fuels.^30^ Figure 4 highlights the biofuel types according to feedstock generation.

Figure 4, classifying biofuels into generations based on their feedstock sources, highlighting the role of water hyacinth as a resource for greener energy. Bioethanol, produced primarily from crops, such as sugarcane, corn, and cellulosic feedstocks, has garnered attention as a viable and sustainable transportation fuel.^107^^,^^108^ Previous generations of biofuels, such as first-generation ethanol from corn, had inferior yields, typically producing only 400 L of ethanol per hectare and needed a lot of feed to create a good amount of biofuels, which can lead to food scarcity as feedstocks are not from non-food crops as compared to the potential of third-generation biofuels from algae, which can yield over 1,000 L per hectare.^39^^,^^109^^,^^110^^,^^111^

Similarly, biodiesel, typically sourced from vegetable oils and animal fats, offers an eco-friendly alternative to diesel fuel, boasting reduced greenhouse gas emissions and superior lubricating properties.^112^ Biofuels encompass various renewable energies derived from biological sources, each with unique properties and applications. Water hyacinth is primarily considered a second-generation biofuel feedstock due to its non-food biomass nature. However, research is exploring its potential in integrated systems that could contribute to third and fourth-generation biofuel production.

Biofuels are renewable fuels derived from organic matter, offering a sustainable alternative to fossil fuels and contributing to a circular economy.^68^^,^^113^ They can be broadly classified based on their production methods and feedstock. Each generation offers unique advantages and disadvantages regarding resource utilization, environmental impact, and economic viability. Understanding these distinctions is crucial for evaluating the sustainability and scalability of water hyacinth-based biofuel production.

### Catalytic conversion efficiency

The catalytic conversion processes employed in this study demonstrated significant efficiency improvements. Enzyme-assisted hydrolysis increased sugar release by 50%, while metal-organic framework (MOF) catalysts achieved a remarkable 90% conversion rate for bio-oil production.^81^^,^^114^ MOF catalysts exhibited superior stability and recyclability compared to traditional thermochemical processes, reducing the frequency of catalyst replacement and associated costs.^115^ A comparative thermal degradation analysis revealed that MOF-assisted pyrolysis required lower activation energy, optimizing energy inputs while improving overall conversion yields. Furthermore, incorporating green catalysts minimized secondary waste generation, contributing to a more sustainable biofuel production process. These findings are consistent with recent advancements in catalyst design for biofuel applications^116^^,^^117^

Catalysis serves as a critical enabler in transforming water hyacinth into value-added biofuels and bio-based products within circular bioeconomy systems. Given the plant’s high moisture and lignocellulosic content, conventional processing methods often face challenges in breaking down fibrous structures efficiently. Enzyme-assisted hydrolysis has proven particularly effective, increasing fermentable sugar release by 50%, which directly enhances ethanol yields. Furthermore, the application of MOF catalysts has significantly improved the pyrolytic conversion of water hyacinth to bio-oil, achieving a 90% conversion efficiency.^81^ These catalysts also demonstrated enhanced thermal stability and reusability, making them economically and environmentally viable for scalable biorefinery operations.

Importantly, these catalytic advancements reduce both energy input and secondary waste, aligning with the principles of circular bioeconomy by maximizing output and minimizing ecological footprint. Thus, catalysis is not just a technical enhancement; it is a cornerstone for making water hyacinth-based biorefineries both feasible and sustainable.

### Carbon-negative potential

The carbon sequestration potential of water hyacinth-based biofuels was quantified through field trials and carbon balance simulations. Results indicate that large-scale implementation of water hyacinth refineries could offset up to 2.5 metric tons of CO_2_ per hectare annually.^118^^,^^119^ Biochar application further enhanced long-term carbon storage while reducing methane emissions from wetland areas previously dominated by decaying water hyacinth biomass.^120^ These findings underscore the potential of water hyacinth-based biofuels as net-carbon-negative solutions for climate change mitigation.^18^^,^^121^ The integration of biochar into soil management practices not only improved soil health but also contributed to atmospheric CO_2_ reduction, aligning with global climate goals.^122^^,^^123^^,^^124^ Table 4 presents a comparative soil parameters based on recent research findings. The data highlight the impacts of biochar on soil carbon sequestration, methane emission reduction, and soil pH, demonstrating its potential benefits for soil health and climate mitigation.

**Table 4:** Carbon sequestration potential of water hyacinth biochar

Parameter	Control soil (no biochar)	Soil with biochar	% Change	Source (s)
Biochar application rate (tons/ha)	0	5–20 (Most studies fall within this range; higher application rates not always economically viable.)	N/A	Balasopoulou et al.^11^; Schenk et al.^125^; Sasidharan et al.^126^
Soil carbon sequestration (tons C/ha/yr)	0.2–0.7 (Baseline depends heavily on soil type, land management practices, and climate.)	1.2–3.5 (Higher values depend on biochar feedstock, pyrolysis conditions, and soil type.)	+200 to +400 (increase)	Mikhailova et al.^127^; Kanwal et al.^128^
Methane emission reduction (%)	Varies significantly depending on wetland type and baseline methane flux.	15-55 (Biochar reduces methane emissions by altering soil microbial communities and redox potential.)	−15 to −55 (reduction)	Ji et al.^129^; Raj et al.^130^
Soil pH change	5.5–7.0 (Optimal range for most agricultural soils.)	6.5–8.0 (Biochar typically increases pH, which can be beneficial for acidic soils.)	+0.5 to +1.5 units (increase)	Guoet al.^131^; Guo et al.^132^; Sriphirom et al.^133^

Table 4 provides a summary of the carbon sequestration potential of water hyacinth biochar compared to control soil without biochar, highlighting key parameters, such as application rates, soil carbon sequestration rates, methane emission reductions, and soil pH changes. The carbon sequestration potential of water hyacinth-based biofuels was quantified through field trials and carbon balance simulations.

Pilot-scale operations for converting water hyacinths into useful products have shown promising results in various regions, including India and Kenya. These operations typically achieve a conversion efficiency of 45–55%, although enzyme deactivation can reduce output by approximately 8% over time.^134^^,^^135^ The energy consumption for these processes is recorded at 2.8–3.2 MJ/kg, with around 11% losses attributed to contamination and downtime, indicating that large-scale implementation of water hyacinth biofuel refineries could offset up to 2.5 metric tons of CO_2_ per hectare annually.^135^^,^^136^^,^^137^

## Discussion

### Water Hyacinth’s biofuel potential

Water hyacinth exhibits exceptional promise for socioeconomic opportunities, such as feedstock for biofuel, its high biomass production, quick growth rates, and capacity to adapt to many environmental circumstances.^16^^,^^138^^,^^139^ By lowering carbon emissions, using water hyacinth to produce biofuels can help achieve sustainability goals, sequestering atmospheric carbon and offering potential benefits for biodiversity.^20^^,^^140^^,^^141^ The findings reveal its potential as a sustainable and economically viable feedstock, aligning with global efforts for renewable energy and climate change mitigation.^142^ Transforming this invasive species into a valuable resource offers a comprehensive approach to tackling both environmental and energy challenges.

### Techno-economic viability and sensitivity analysis

The LCOE for water hyacinth biofuel was lower than that of second-generation biofuels, reducing production costs by approximately 25%, indicating a viable renewable energy source. Decentralized processing units reduced transportation costs, enhancing localized supply chains.^61^^,^^143^ The compositional analysis (Figure 3) highlights water hyacinth’s potential as resources for biofuels.^70^^,^^84^ Sensitivity analysis shows that a 15% fluctuation in enzyme prices results in a ±10% variation in LCOE.^144^ Seasonal biomass yield variation can shift unit costs by up to 18%, highlighting the need for storage strategies or mixed feedstock use. Its rapid growth and abundance provide a 30% cost advantage as a cost-effective biofuel feedstock.^145^ This lowers environmental management costs, increases energy security and establishes water hyacinth as a contender in the global biofuel market, especially in abundant regions.^146^ However, challenges remain in scaling up production and ensuring consistent feedstock supply throughout the year.

### Advances in genetic and metabolic engineering

Genetic engineering approaches have demonstrated significant improvements in bioethanol production and enzymatic hydrolysis efficiency.^147^ Experimental results showed a 40% increase in bioethanol production and a 20% enhancement in enzymatic hydrolysis efficiency, reducing fermentation time.^66^^,^^147^ These findings align with recent research highlighting the role of genetic modifications in optimizing lignocellulosic biomass for biofuel production.^148^^,^^149^

Metabolic engineering further facilitated the introduction of high-yield fermentation pathways, significantly improving sugar conversion rates. Comparative studies with conventional enzymatic treatments revealed that genetically optimized strains exhibited a 15% higher efficiency in breaking down complex polysaccharides, accelerating bioethanol production.^66^ These advancements are consistent with research on fourth-generation biofuels, where targeted genetic modifications have been shown to substantially increase sugar release and biofuel yields. These improvements position water hyacinth as a competitive feedstock for bioethanol production while highlighting the need for continuous R&D to mitigate potential ecological risks associated with genetically modified species.^123^^,^^150^

### Multi-product biorefinery and environmental sustainability

A multi-product biorefinery model improves economic viability and promotes environmental sustainability. Resource utilization is maximized, and waste is reduced by using co-products like biochar and biogas.^20^^,^^54^^,^^113^^,^^151^ Biochar application improved carbon sequestration and reduced soil acidity by 15%, with additional effects on soil properties and carbon storage.^152^ Building upon bioethanol production, the study explored the potential for generating valuable co-products such as biochar and biogas.^153^ The integration of biochar application into agricultural soil demonstrated a dual benefit improving carbon sequestration while reducing soil acidity by 15%. Notably, biogas production efficiency was increased by 30% supporting the viability of a multi-product biorefinery approach.

Few LCA studies address aquatic toxicity as zooplankton after intensive harvesting observed 13% decline.^92^ Hence, integrating aquatic species loss and plankton diversity indices (e.g., Shannon-Wiener Index) into LCA.^77^^,^^154^ Incorporating such metrics is critical for validating sustainability claims of large-scale harvesting interventions.

Results demonstrated that enzyme-assisted hydrolysis enhanced sugar release by 50%,^155^^,^^156^ while the application of MOF catalysts achieved a bio-oil conversion rate of 90%, outperforming traditional thermochemical processes.^157^ Recent studies have confirmed the effectiveness of enzyme-assisted methods in breaking down complex polysaccharides, significantly improving the yield of fermentable sugars for bioethanol production.^158^^,^^159^ Similarly, MOF catalysts have been shown to optimize catalytic activity due to their high surface area and hierarchical pore structures, enhancing bio-oil production efficiency^97^^,^^160^

The utilization of MOF catalysts also demonstrated improved stability and recyclability, reducing the need for frequent catalyst replacement.^155^ Research indicates that MOFs can significantly enhance catalytic performance and longevity in biomass conversion processes, making them a cost-effective solution for large-scale applications.^157^ A comparative thermal degradation analysis further highlighted that MOF-assisted pyrolysis exhibited a lower activation energy requirement, optimizing energy input and improving overall conversion yields.^155^

Moreover, the incorporation of green catalysts minimized secondary waste production, contributing to a more sustainable biofuel production process.

The carbon sequestration potential of water hyacinth biofuels reveals significant methane emission reductions. Large-scale water hyacinth biofuel refineries have the capacity to offset up to 2.5 metric tons of CO_2_ per hectare annually, as evidenced by studies and simulations.^161^^,^^162^^,^^163^ Research indicates that water hyacinth can sequester substantial amounts of carbon dioxide in wetlands, while also producing organic matter that can enhance soil quality.^164^ Through soil management, biochar is integrated to improve carbon storage and lower methane emissions from wetland areas, making water hyacinth-based biofuels a net-carbon-negative solution.

### Socio-environmental impacts and trade-offs

Cross-sectoral impacts include potential job creation in harvesting and processing units, particularly for marginalized communities. However, this must be balanced with ecosystem services trade-offs.^165^ LCA findings from select studies indicate potential reductions in carbon intensity by 15–30%, yet socio-environmental trade-offs remain underexplored in low-income regions.

Biofuel interventions have demonstrated socioeconomic co-benefits, such as employment generation, gendered labor inclusion, and rural electrification.^43^ However, in regions with weak institutional support, these benefits may remain uneven. Incorporating water hyacinth biofuel into a circular economy requires attention to social trade-offs, such as loss of artisanal fishing access due to biomass extraction. These interdependencies in community-centric bioenergy transitions suggest that local co-ownership models can improve outcomes.^166^^,^^167^

The application of biochar derived from water hyacinth has been shown to enhance long-term carbon storage and mitigate methane emissions effectively.^18^^,^^66^ This is significant; however, it needs to be carefully addressed because the stability of biochar and its possible consequences for long-term carbon storage must be carefully assessed. This study evaluated anaerobic digestion, fermentation, and transesterification conversion technologies, each with distinct advantages and disadvantages. Factors affecting biogas production and digester parameters make anaerobic digestion a feasible option.^168^ Effective pre-treatment methods are needed to optimize sugar release for fermentation procedures.^47^^,^^97^ Because of the low lipid content of water hyacinth, transesterification is still doable when paired with lipid extraction techniques.^169^ To maximize resource utilization and sustainability, conversion techniques must be customized.

### Ecological risks and mitigation strategies

Water hyacinth valorization offers economic and ecological benefits, but unchecked biomass harvesting can disrupt habitats. Mitigating risks, regulatory controls, regular ecological monitoring, and seasonal peak harvesting prioritization are essential. Exploring less invasive native alternatives like Lemna minor or Azolla can also be beneficial, particularly in fragile aquatic ecosystems.^170^ The importance of sustainable practices, such as early detection protocols, mechanical containment, and periodic ecosystem health audits, to prevent biodiversity losses and microbial imbalances.^99^

### Scalability, land use, and policy integration

For scalability, decentralized modular units can be deployed in lake-adjacent communities. Integration with local energy grids requires policy interventions for feed-in tariffs and subsidies. Land-use impact assessments show negligible competition with food crops due to aquatic nature; however, large-scale processing facilities might induce land conversion risks if not regulated. Scalability can be enhanced via modular biorefinery units,^53^ which lower entry barriers in rural contexts. Integrating into regional grids, as in Kenya, requires regulatory incentives and microfinancing.^101^

While water hyacinth avoids direct food-fuel conflicts, its influence on shoreline land use and local labor reallocation may affect seasonal farming systems. Though aquatic cultivation reduces direct land competition, indirect effects such as reallocation of community labor and use of shoreline land for processing facilities could influence local agricultural practices.^14^

Using water hyacinth to produce biofuel is consistent with global efforts to achieve the sustainable development goals (SDGs), most notably SDG 7 (affordable and clean energy) and SDG 15 (life on land).^3^^,^^127^^,^^171^ 64% of the studies reviewed originated from Asia (India and China), 23% from Africa (Kenya and Nigeria), and less than 5% from South America and Europe. This regional disparity suggests a potential limitation in generalizability.^172^ Hence, Latin America, being the native range, is underrepresented in scalable implementation studies, possibly due to socio-political or institutional constraints. This strategy promotes sustainable waste management practices and ecosystem restoration by turning an invasive species into a useful resource. India, Nigeria, Bangladesh, and Kenya are among the countries looking into a variety of uses for water hyacinths, including biogas production, biodiesel production, and handicraft production.^40^^,^^41^^,^^44^^,^^173^^,^^174^ LCA from select pilot studies reported a 12–18% reduction in plankton diversity and 9% benthic disturbance following intensive harvesting. Inclusion of ecological toxicity metrics, such as LC50 and biodiversity indices, is essential in future assessments.

### Limitations and future research directions

Despite the promising potential of water hyacinth as a bioenergy feedstock, several limitations constrain its large-scale utilization. Limitations of this review include a strong geographic bias in available studies, with most research concentrated in Asia and sub-Saharan Africa, while Latin America, Europe, and North America remain underrepresented despite their ecological and policy relevance. Additionally, most evidence is derived from laboratory or small-scale studies, with minimal industrial-scale validation. This creates uncertainty around scalability, process reliability, enzyme deactivation, and cost fluctuations in real-world conditions. Furthermore, ecological risks associated with large-scale biomass harvesting, such as aquatic biodiversity loss, benthic disruption, and microbial imbalance are seldom included in LCA, despite their importance for long-term ecosystem integrity.

Future research should prioritize industrial-scale trials to test system performance under varied environmental and policy contexts. Standardized frameworks for integrating ecological toxicity and biodiversity metrics (e.g., plankton diversity indices, benthic health indicators) into LCA are urgently needed. Cross-country policy analysis, particularly in underrepresented regions, will support a more global understanding of feasibility. Finally, participatory governance models that incorporate community ownership, gendered labor dynamics, and energy justice considerations will be essential for ensuring equitable, sustainable, and resilient transitions to circular bioeconomy systems rooted in water hyacinth valorization.

### Conclusion

A systematic review highlights the immense potential of water hyacinth as a sustainable biofuel feedstock, offering economic, environmental, and energy security benefits. By synthesizing fragmented research, this study provides the most comprehensive assessment to date, addressing critical gaps in techno-economic feasibility, conversion efficiency, and policy frameworks. The findings demonstrate that water hyacinth biofuels can achieve lower LCOE, higher bioethanol yields (40% increase through genetic modifications), and enhanced sustainability through multi-product biorefinery models. Additionally, its carbon-negative potential, offsetting up to 2.5 metric tons of CO_2_ per hectare annually, presents a compelling climate mitigation strategy. However, scaling water hyacinth biofuel production remains a challenge due to gaps in life cycle assessments, feedstock supply chain stability, and regulatory frameworks for decentralized biorefineries. Future research must prioritize large-scale pilot studies, improved enzymatic hydrolysis and MOF catalyst efficiency, and the development of policy-driven incentives to enhance commercial viability. Addressing these gaps through interdisciplinary collaborations and sustainable investment strategies will be crucial to unlocking water hyacinth’s full potential in the global bioeconomy. Transforming this invasive species into a valuable energy resource aligns with international sustainability goals and circular economy principles, paving the way for its integration into global renewable energy policies.

## Acknowledgments

Deep gratitude is extended to the E4LIFE International Ph.D. Fellowship Program, generously supported by 10.13039/100009526Amrita Vishwa Vidyapeetham, for making this research possible. Heartfelt thanks go to the Amrita School for Sustainable Futures and the Amrita Live-in-Labs academic Program for their unwavering encouragement and guidance. Their support has been instrumental in this success, and it is an honor to have had the chance to work alongside such a dedicated and visionary team.

## Author contributions

A.A., writing the original draft; R.A.M., visualized the data; S.S., supervision, review, and editing

## Declaration of interests

The authors declare no competing interests.
